# A Single-Nucleotide Polymorphism of *TaGS5* Gene Revealed its Association with Kernel Weight in Chinese Bread Wheat

**DOI:** 10.3389/fpls.2015.01166

**Published:** 2015-12-23

**Authors:** Shasha Wang, Xiangfen Zhang, Feng Chen, Dangqun Cui

**Affiliations:** Agronomy College, National Key Laboratory of Wheat and Maize Crop Science, and Collaborative Innovation Center of Henan Grain Crops, Henan Agricultural UniversityZhengzhou, China

**Keywords:** bread wheat, *TaGS5* gene, yield-related traits, kernel size, RT-PCR

## Abstract

*TaGS5* genes were cloned from bread wheat and were physically mapped on 3AS and 3DS. Sequencing results revealed that a SNP was found in the sixth exon of *TaGS5-A1* gene. The SNP resulted in amino acid change from alanine to serine at the 303 bp position of *TaGS5-A1*. These two alleles were designated as *TaGS5-A1a* (alanine at the 303 bp position) and *TaGS5-A1b* genes (serine at the 303-bp position). Analysis of association of *TaGS5-A1* alleles with agronomic traits indicated that cultivars with *TaGS5-A1b* possessed wider kernel width and higher thousand-kernel weight, as well as significantly lower plant height, spike length, and internode length below spike than those of cultivars with *TaGS5-A1a* over 3 years. These trait differences between *TaGS5-A1a* and *TaGS5-A1b* genotypes were larger in landraces than in modern cultivars. This finding suggested that *TaGS5* gene played an important role in modulating yield-related traits in the landraces, which possibly resulted from numerous superior genes gathering in modern cultivars after strong artificial selection. The preferred *TaGS5-A1b* haplotype underwent very strong positive selection in Chinese modern wheat breeding, but not in Chinese landraces. Expression analysis of the *TaGS5-A1* gene indicated that *TaGS5-A1b* allele possessed significantly higher expression level than *TaGS5-A1b* allele in differently developmental seeds. This study could provide relatively superior genotype in view of agronomic traits in wheat breeding programs. Likewise, this study could offer important information for the dissection of molecular and genetic basis of yield-related traits.

## Introduction

Wheat is an important food crop in the world, and improvement of wheat yield has always been an essential breeding target for wheat breeders over the past years. As one of the three wheat yield elements, thousand-kernel weight (TKW) is considered to have an important influence on wheat yield and could be determined by the kernel size. To date, some important yield-related genes (GS3, GS5, GW2, GW3, GW5, GW6, GW8, GIF1, and Ghd7) associated with kernel size were gradually discovered and cloned in rice (Li et al., 2004a,b, 2011; Fan et al., 2006; Xie et al., 2006; Song et al., 2007; Wang et al., 2008, 2012b; Weng et al., 2008; Xue et al., 2008; Guo et al., 2009; Takano-Kai et al., 2009, 2013). For example, the *GS5* gene, encoding a putative serine carboxypeptidase, was cloned in rice as a positive regulator of grain size by regulating grain width, filling, and weight (Li et al., 2011). More recently, *ZmGS5* gene also were cloned by *in silico* cloning and was found to be associated with kernel weight and cell number in maize (Liu et al., 2015). Meanwhile, some co-orthologs associated with kernel size and weight were successfully cloned in maize (Li et al., 2010). These factors facilitated the cloning of genes related to kernel size in polyploidy wheat.

Hexaploid wheat has a larger genome size (≈17.9 Gb) compared with rice (≈400 Mb) and maize (≈3 Gb; Varshney et al., 2006). Therefore, direct cloning of yield-related genes from hexaploid wheat without reference genes was difficult. However, a large number of yield-related quantitative trait loci (QTLs) were identified in polyploid wheat. These QTLs were mapped on almost all of the chromosomes, including group 3 in bread wheat (Varshney et al., 2000; Wang et al., 2009; Zhang et al., 2010, 2012; Wu et al., 2012; Cui et al., 2014; Liu et al., 2014). Moreover, a number of yield-related genes were cloned from polyploid wheat based on *in silico* cloning. Wheat sucrose synthase two orthologous gene (*TaSus2*) was isolated and found to be significantly associated with TKW (Jiang et al., 2011). Two putative cytokinin oxidase genes (*TaCKX2.1* and *TaCKX2.2*) were cloned and showed intimate relationship to kernel number per spike of bread wheat (Zhang et al., 2011). A cell wall invertase gene *TaCwi-A1* could explain 4.8% of phenotypic variance for kernel weight over 2 years (Ma et al., 2010). A *TaGW2* gene obtained by *in silico* cloning was significantly associated with TKW, heading, and maturity in bread wheat (Su et al., 2010; Hong et al., 2014; Simmonds et al., 2014). *TaGW2* also negatively regulated kernel weight by controlling gene expression level during seed development (Qin et al., 2014).

In this study, we successfully obtained *TaGS5* genes from A and D genomes of bread wheat by *in silico* cloning and mapped them on the short arm of chromosomes 3A and 3D. Analysis of association with agronomic traits indicated that wheat cultivars with *TaGS5-A1b* allele possess relatively preferred agronomic traits. The purpose of this study is to illustrate association of allelic variation of *TaGS5-A1* gene with agronomic traits and uncover the relatively superior *TaGS5-A1* alleles in view of agronomic traits in order to provide useful information for the selection of relatively preferable genotype in view of kernel size and plant height in wheat breeding program.

## Results

### Cloning of *TaGS5* gene in bread wheat

Based on the cDNA sequence of *OsGS5* in rice, a number of expressed sequence tags (ESTs) were collected from transcriptomes of durum and bread wheat cultivars, as well as NCBI database by full alignment. A putative full-length *TaGS5* cDNA sequence was generated by assembling above ESTs. Furthermore, a 1446 bp cDNA sequence was successfully amplified in the cDNA of Chinese Spring with primer set TaGS5_−_P1 (Table 1), which was designed according to the above-mentioned putative *TaGS5* cDNA sequence.

**Table 1:** **Primers used for the identification of *TaGS5* gene in this study**.

**Primer**	**Primer sequence (5′–3′)**	**Annealing temperature (°C)**	**PCR fragment size (bp)**
TaGS5-P1	Forward: CAAGCCACTCACTCTCACAT Reverse: TCCTTGAACTCATTTTGGGTCA	57	1533
TaGS5-P2	Forward: CAAGCCACTCACTCTCACAT Reverse: GATCAGCGCTATCCCTTCTG	57	1436
TaGS5-P3	Forward: AGCCACTCACTCTCACATTTG Reverse: AGAAGGAATGTGTCGATCAGC	58	1448
TaGS5-P4	Forward: AGCCAAGCCACTCACTCT Reverse: CTCTCCTTGAACTCATTTTGGG	56	1504
TaGS5-P5	Forward: GCGAACCAAGACAAGCAG Reverse: CCTTGTACTGCGGAAACCTC	56	930
TaGS5-P6	Forward: CTTCTGAGCTAGGACCTCTC Reverse: ACAAGGTCAGCTAGTTGTGG	56	1226
TaGS5-P7	Forward: ACATCCTCTGACCTCACCAA Reverse: GATACAACTGCATGGCTCCA	57	1427
TaGS5-P8	Forward: TCATTATGTGCCACAACTAGCT Reverse: AGTACCGAAAAGTTGTACGACT	57	1225
TaGS5-P9	Forward: TGTCAATGGGATGTTGCCTG Reverse: TCATCGGTGTGTAGGAAGCTG	58	1162
TaGS5-P10	Forward: TCATACACACATAATCCAGTCGA Reverse: GATCGTGGGTGTTGCATCTAT	55	800
TaGS5-P11	Forward: GACTTAGAACCACGACAGCC Reverse: CGTAGCATCCATCGGCATG	57	1086
TaGS5-P12	Forward: GAGCACAAGAGTGAAGCGAGATGG Reverse: CGTTGTTGGCGTATGCGTCTGA	59	1400
TaGS5-P13	Forward: AAGGTCGGGCAAAGTCTATG Reverse: CGAGGAGAAAGAGAGCAAGGA	56	1000
TaGS5-P14	Forward: GAAGGCCAGCACATACATCA Reverse: TGTGCCACCTGTCATTTCTT	57	2127
18s	Forward: CCTGCGGCTTAATTGACTC Reverse: GTTAGCAGGCTGAGGTCTCG	56	150
TaGS5-P15	Forward: GAAGGCCAGCACATACATCA Reverse: GCTGCTGATGTTTGTCCA	56	276
TaGS5-P16	Forward: TAGAGCCTCAAACTGGACCG Reverse: AGATGCTGATGATGTTTGTCCA	56	127

To further obtain full-length *TaGS5* genomic DNA sequence, five primer sets (TaGS5_−_P5 to TaGS5_−_P9) were designed according to *TaGS5*cDNA sequence. Exon positions were deduced by full alignment of *TaGS5* cDNA sequence and Os*GS5* gDNA sequence. Five corresponding fragments were successfully amplified in gDNA of Chinese Spring with primer sets TaGS5_−_P5 to TaGS5_−_P9. A total of 70 subclones were sequenced from both directions after ligation with T-Easy vector. Genomic DNAs of *T. urartu, Ae. speltoids, T. tauschii*, and durum wheat were used to map the genomic location of each subclone. Based on the positioned sequences of subclones, two full-length *TaGS5* gDNA sequences were successfully assembled on A and D genomes of Chinese Spring and were designated as *TaGS5-A1* and *TaGS5-D1* genes (Figure S1).

**Figure 1:**
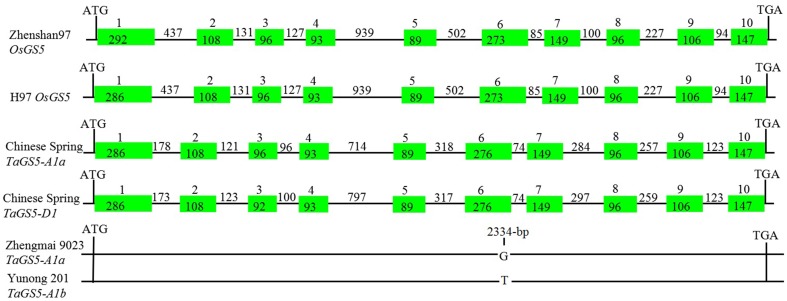
**Schematic representation of the structures of the TaGS5 and OsGS5 genes in bread wheat and rice**. Filled boxes and lines represent exons and introns, respectively.

Physical mapping by a set of nullisomic–tetrasomic lines and ditelosomic lines of Chinese Spring indicated that *TaGS5-A1* and *TaGS5-D1* genes were located on the short arm of chromosomes 3A and 3D, respectively. Sequencing blast of *TaGS5-A1* and *TaGS5-D1* genes in URGI database of Chinese Spring (http://wheat-urgi.versailles.inra.fr/) indicated that *TaGS5-A1* gene is identical to contig TA_454_ch3A|scaffold21314_length_7500 on the chromosome 3A, whereas *TaGS5-D1* gene is identical to contig IWGSC_3DS|IWGSC_chr3DS_ab_k95_contigs_longerthan_200_2595062 on the chromosome 3DS. This finding suggested that the sequences and chromosome locations of *TaGS5-A1* and *TaGS5-D1* genes were very reliable. Further analysis of gDNA sequences of *TaGS5-A1* with 3611 bp and *TaGS5-D1* with 3705 bp indicated that both *TaGS5-A1* and *TaGS5-D1* genes were composed of 10 exons and 9 introns (Figure 1). The deduced amino acid sequence showed that both *TaGS5-A1* and *TaGS5-D1* genes could encode a 481 aa protein with six domains as predicted using software SMART (http://smart.embl-heidelberg.de/). These domains were alpha amylase inhibitor domain at 22–74 interval, putative carbohydrate binding domain at 102–163 interval, RIIalpha (regulatory subunit portion of type II PKA R-subunit) domain at 153–183 interval, fibrinogen-related (FBG) domain at 234–383 interval, Bowman–Birk type proteinase inhibitor domain at 260–296 interval, and phosphoinositide 3-kinase region postulated to contain C2 domain at 286–422 interval.

### Molecular characterization of *TaGS5* genes in chinese bread wheat

**Figure 2:**
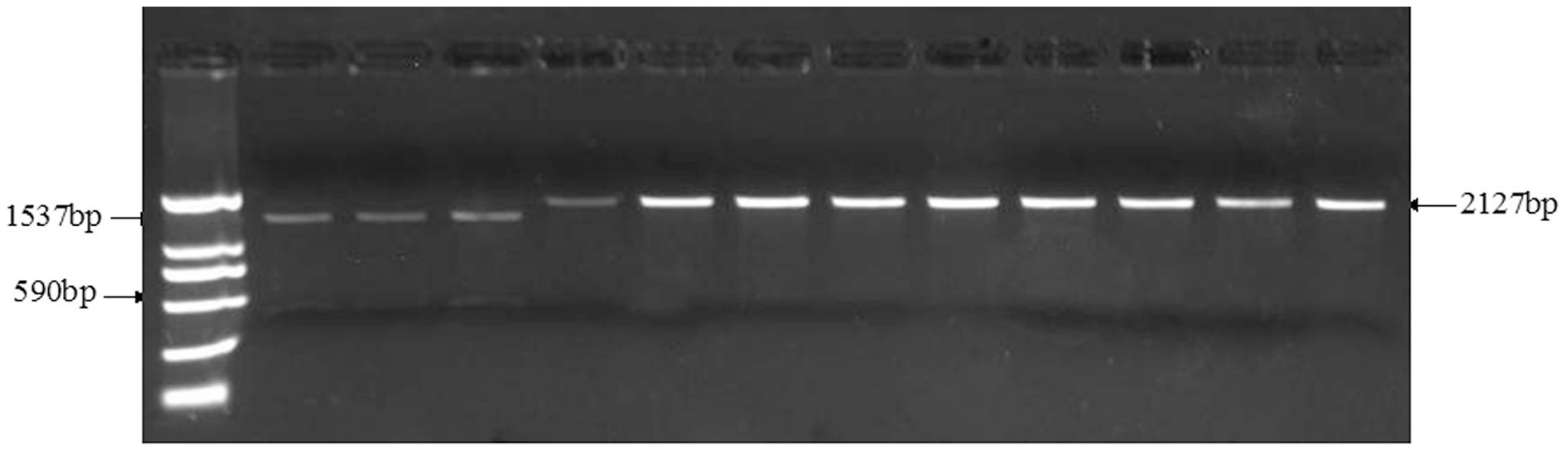
**Identification of *TaGS5-A1a* and *TaGS5-A1b* alleles by digestion of restriction enzyme *Bbv1***.

Four primer sets (TaGS5_−_P10 to TaGS5_−_P13, Table 1) were used to analyze nucleotide polymorphism of *TaGS5-A1* promoter sequence in the 90 selected wheat cultivars. No fragment difference was found in the promoter of *TaGS5-A1* gene. Furthermore, 20 wheat cultivars, which were composed of 10 cultivars with relatively large kernel size, and 10 cultivars with relatively small kernel size, were selected to sequence cDNA sequences of *TaGS5-A1* and *TaGS-D1* genes amplified with primer set TaGS5_−_P1 and TaGS5_−_P10–P13 (Table 1). Sequencing results revealed that a SNP of G/T was found at position 907 bp of cDNA sequences (Figure 2), which was in the predicted FBG domain of *TaGS5-A1* gene. Furthermore, five primer sets (TaGS5_−_P5–P9, Table 1) were used to identify allelic variations in *TaGS5-A1* and *TaGS5-D1* genomic DNA sequences among all surveyed cultivars, but no other polymorphism was found. Further analysis of the SNP indicated that the G/T mutation occurred at position 2334 bp in the sixth exon of *TaGS5-A1* genomic DNA sequence. Sequencing results confirmed the reliability of the SNP. This SNP resulted in an amino acid change from alanine to serine. The *TaGS5-A1* allele with G at position 907 bp of *TaGS5-A1* cDNA sequence was designated as *TaGS5-A1a*, whereas the *TaGS5-A1* allele with T at position 907 bp of *TaGS5-A1* cDNA sequence was designated as *TaGS5-A1b* according to the nomenclature of McIntosh et al. (2007).

The alternation of G to T (GCAGC to TCAGC) in *TaGS5-A1* gene broke the digestion site of restriction enzyme *Bbv1* (GCAGC). Therefore, restriction enzyme *Bbv1* was used to digest 2127 bp fragments amplified with primer set TaGS5_P13 (Table 1) in different wheat cultivars to distinguish *TaGS5-A1a* and *TaGS5-A1b* alleles. After digestion by *BbvI*, cultivars with both 590 and 1537 bp fragments possessed *TaGS5-A1a* allele, and cultivars with only 2127 bp fragment possessed *TaGS5-A1b* allele (Figure 2).

### Association between *TaGS5-A1* alleles and kernel size and other agronomic traits

Based on the results of PCR amplification with primer set TaGS5_P14 and digestion by enzyme *BbvI*, 15 and 26 out of 41 landraces, respectively, belonged to *TaGS5-A1a* and *TaGS5-A1b* alleles, and 70 and 252 out of 322 modern cultivars belonged to *TaGS5-A1a* and *TaGS5-A1b* alleles, respectively. Variance analysis in the surveyed landraces indicated that plant height, panicle length, and internode length below spike of cultivars with *TaGS5-A1a* alleles were relatively higher than those with *TaGS5-A1b* alleles. Moreover, TKW, kernel length, and kernel width of wheat landraces with *TaGS5-A1b* alleles were significantly higher than those of wheat cultivars with *TaGS5-A1a* alleles over 3 years. The grain length/grain width (GL/GW) ratio of landraces with *TaGS5-A1a* was also significantly higher than that of cultivars with *TaGS5-A1b* over 3 years (Table 2).

**Table 2:** **Comparison of agronomic traits of bread cultivars with *TaGS5-A1a* and *TaGS5-A1b* alleles**.

	**Trait**	**2013**		**2014**		**2015**	
		***TaGS5-A1a***	***TaGS5-A1b***	***TaGS5-A1a***	***TaGS5-A1b***	***TaGS5-A1a***	***TaGS5-A1b***
	Sample number	85	278	85	278	85	278
	Plant height (cm)	81.39a	75.64a	96.58A	77.72B	100.25A	92.19B
	Spike length (cm)	10.24a	9.81b	10.02A	9.14B	10.90a	10.38b
	Internode length below spike (cm)	27.23A	25.04B	31.62a	29.75a	32.77A	30.30B
	Spikelet number per spike	19.03a	19.35a	19.56a	19.88a	19.11a	19.32a
Total	Kernel number per spike	44.82a	43.00a	51.56a	53.07a	48.26a	48.09a
	Kernel length (mm)	6.89a	6.85a	7.00a	6.93a	6.93a	6.81a
	Kernel width (mm)	3.22B	3.29A	3.46a	3.72a	3.38a	3.42a
	Kernel length/kernel width ratio	2.15A	2.09B	2.03A	1.93B	2.06a	2.00b
	Thousand-kernel weight (g)	40.86b	42.94a	49.24a	50.72a	41.52a	42.52a
	Sample number	15	26	15	26	15	26
	Plant height (cm)	103.89a	92.49a	117.00A	98.44B	123.66A	103.36B
	Spike length (cm)	10.25a	9.90a	10.33a	9.94a	10.87a	10.35a
	Internode length below spike (cm)	31.98a	30.19a	40.10a	34.35b	37.14a	34.81a
	Spikelet number per spike	18.96a	19.44a	19.62a	19.85a	19.64a	19.28a
Landraces	Kernel number per spike	46.76a	41.94a	50.12a	47.37a	50.33a	47.32a
	Kernel length (mm)	6.48b	6.81a	6.53b	6.96a	6.43b	6.73a
	Kernel width (mm)	3.01b	3.16a	3.23B	3.51A	3.21b	3.40a
	Kernel length/kernel width ratio	2.16a	2.16a	2.05a	1.99a	2.01a	1.99a
	Thousand-kernel weight (g)	33.52b	39.07a	40.46B	46.65A	35.73b	41.21a
	Sample number	70	252	70	252	70	252
	Plant height (cm)	76.57a	73.90a	80.06a	75.58b	95.23a	91.04b
	Spike length (cm)	10.24a	9.80b	9.96A	9.06B	10.90a	10.38b
	Internode length below spike (cm)	26.21a	24.51b	29.80a	29.28b	31.83a	29.84b
	Spikelet number per spike	19.04a	19.34a	19.55a	19.89a	18.99a	19.32a
Modern cultivars	Kernel number per spike	44.40a	43.11a	51.87a	53.66a	47.82a	48.12a
	Kernel length (mm)	6.98a	6.85b	7.10A	6.93B	7.03A	6.82B
	Kernel width (mm)	3.26a	3.31a	3.53a	3.75a	3.416a	3.418a
	Kernel length/kernel width ratio	2.15A	2.08B	2.02A	1.92B	2.07A	2.00B
	Thousand-kernel weight(g)	42.44a	43.34a	51.12a	51.14a	42.76a	42.66a

*Uppercase and lowercase letters after numbers showed extreme (P < 0.01) and significant (P < 0.05) differences, respectively*.

Variation analysis in surveyed modern cultivars showed that plant height, spike length, and internode length below spike of cultivars with *TaGS5-A1a* alleles were also relatively higher than those with *TaGS5-A1b* alleles (Table 2). However, the differences between the traits of cultivars with two *TaGS5-A1* alleles sharply reduced in the modern cultivars when compared with the landraces (Table 2). Furthermore, the kernel length and GL/GW ratio of cultivars with *TaGS5-A1a* were significantly higher than that of cultivars with *TaGS5-A1b* over 3 years. Kernel width and TKW of cultivars with *TaGS5-A1b* were slightly higher than those with *TaGS5-A1a* in 2013 and 2014 (Table 2). This finding suggested that *TaGS5-A1b* is a relatively preferred allele in view of agronomic traits in bread wheat. This allele for high TKW probably underwent strong positive selection in Chinese modern wheat breeding because of its high percentage (78.2% in the modern cultivars and 63.4% in the landraces). Additionally, no significant difference existed between the spikelet number per spike and kernel number per spike of the landraces and modern cultivars with *TaGS5-A1a* and *TaGS5-A1b* over 3 years (Table 2).

To further determine the influence of *TaGS5-A1* alleles on kernel size and other agronomic traits in modern cultivars, a F_10_ RIL population was examined using *TaGS5-A1* CAPS marker. Results showed that 64 and 89 out of 153 wheat inbred lines belonged to *TaGS5-A1a* and *TaGS5-A1b* alleles, respectively. The average of each trait of the lines under two locations was used to analyze the association of *TaGS5-A1* alleles with kernel size and other agronomic traits (Table 3). Variance analysis also indicated that plant height (92.2 cm), spike length (11.1 cm), and internode length below spike (39.2 cm) of the lines with *TaGS5-A1a* alleles were significantly higher than those (88.1, 10.07, and 36.6 cm, respectively) of the lines with *TaGS5-A1b* alleles (*P* < 0.05; Table 3). Further analysis indicated that kernel length of lines with *TaGS5-A1b* (7.08 mm) were slightly narrower than that of lines with *TaGS5-A1a* (7.09 mm), and kernel width of lines with *TaGS5-A1b* (3.52 mm) were slightly wider than that of lines with *TaGS5-A1a* (3.47 mm), but these differences were not significant. However, the average of TKW of the lines with *TaGS5-A1b* alleles (45.8 g) was significantly higher than that of the lines with *TaGS5-A1a* alleles (44.9 g; *P* < 0.05). The difference mainly resulted from relatively wider kernel width of the lines with *TaGS5-A1b* allele (Table 3). In addition, the GL/GW ratio of the lines with *TaGS5-A1a* alleles (2.04) was significantly higher than that of the lines with *TaGS5-A1b* alleles (2.01; *P* < 0.05). This result is consistent with the association between *TaGS5-A1* alleles and agronomic traits in landraces and modern cultivars, as mentioned above.

**Table 3:** **Comparison of agronomic traits of lines with *TaGS5-A1a* and *TaGS5-A1b* alleles using the F_10_RIL population of UC 1110/ PI 610750**.

**Trait**	**F_10_ RIL population**	
	***TaGS5-A1a***	***TaGS5-A1b***
Sample number	64	89
Plant height (cm)	92.2a	88.1b
Spike length (cm)	11.1a	10.7b
Internode length below spike (cm)	39.2a	36.6a
Spikelet number per spike	18.1a	18.7a
Kernel number per spike	–	–
Kernel length (mm)	7.09a	7.08a
Kernel width (mm)	3.47a	3.52a
Kernel length/kernel width ratio	2.04a	2.01b
Thousand-kernel weight (g)	44.9b	45.8a

*Lowercase letters after numbers showed and significant (P < 0.05) differences, respectively*.

**Figure 3:**
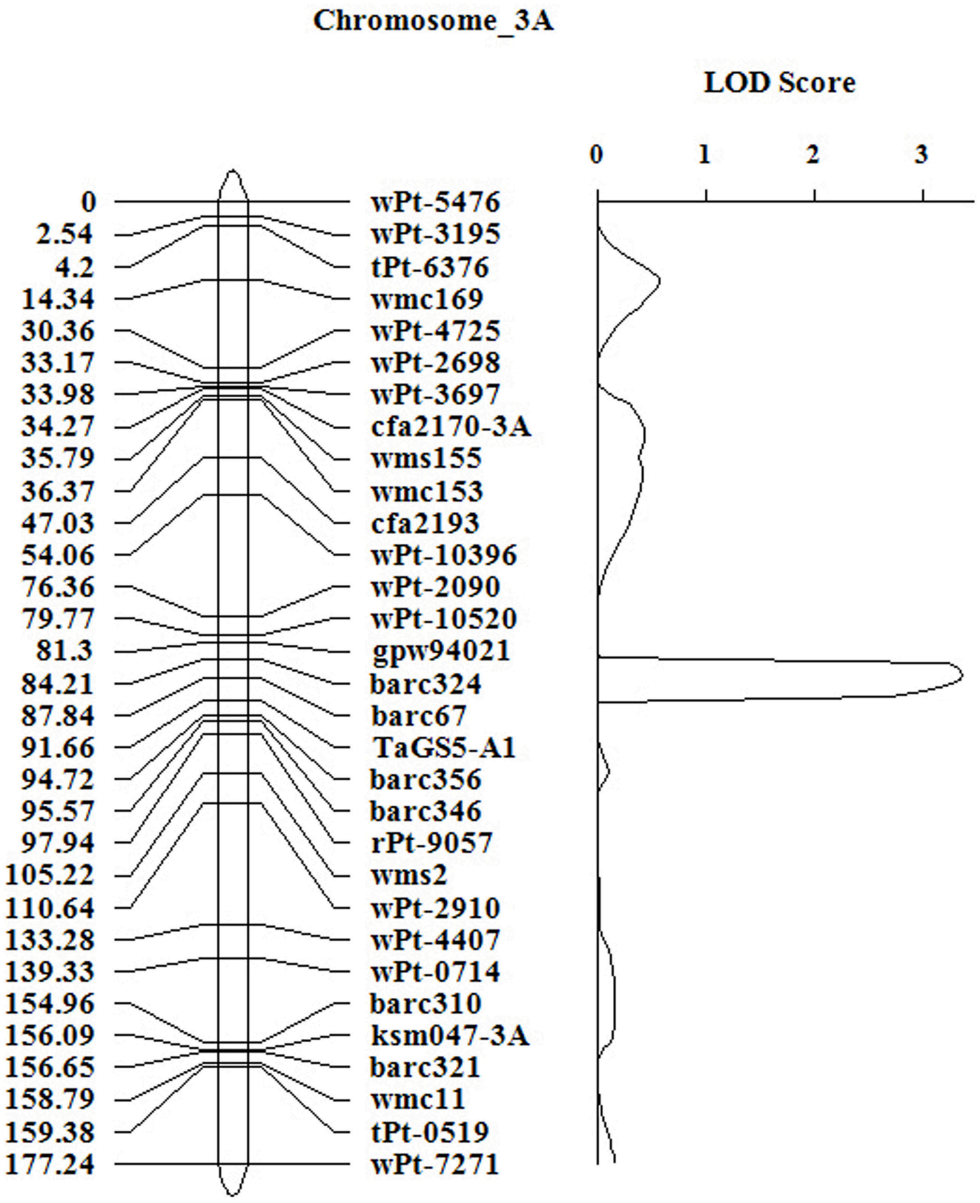
**Linkage map of *TaGS5-A1* gene on 3AS and logarithm of odds contours obtained by composite interval mapping for quantitative trait loci on thousand-kernel weight in the F_10_ RIL population of UC 1110/ PI 610750**. The data of thousand-kernel weight were averaged over two locations.

**Figure 4:**
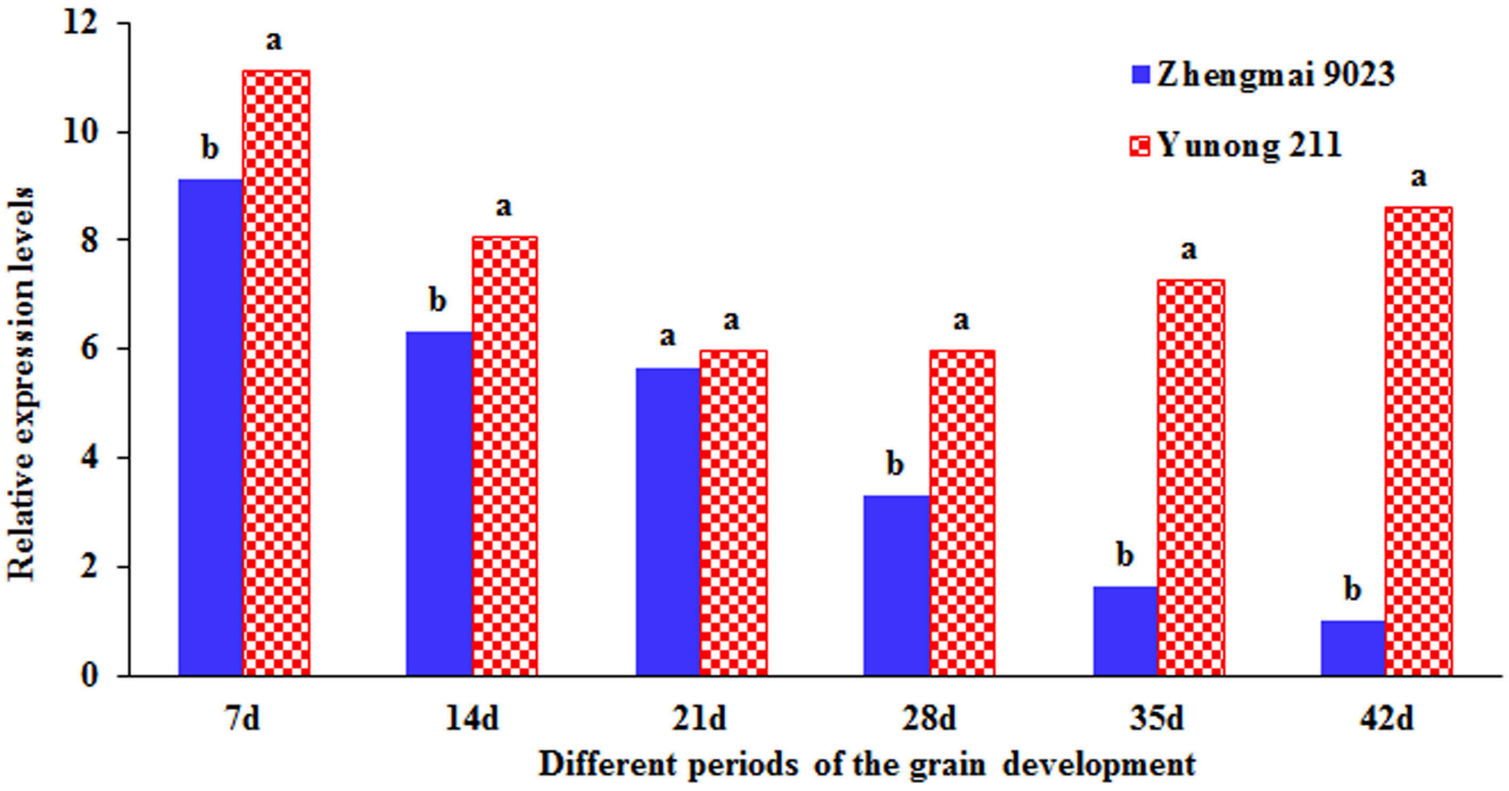
**Relative expression level of *TaGS5-A1* gene in seeds of different developmental stages of Yunong 211 and Zhengmai 9023**. Different letters on the top of the bars indicated the significant difference at 5% probability level.

To confirm the chromosomal location and effect of *TaGS5-A1* gene in bread wheat, *TaGS5-A1* gene was further mapped using the F_10_ RIL population (**Figure 4**). Linkage analysis showed that a major QTL for TKW was mapped on chromosome 3AS, between simple sequence repeat markers *Barc356* and *Barc67* (C-3AS2-0.23). The 8.2% of the phenotypic variance in the F_10_ populations over two environments was explained with a logarithm of odds (LOD) score of 3.36 (Figure 3).

### Expression analysis of *TaGS5-A1a* and *TaGS5-A1b* genotypes

Two cultivars, namely, Zhengmai 9023 with *TaGS5-A1a* and Yunong 211 with *TaGS5-A1b*, were selected to identify expression levels at six different developmental stages of the seeds (Figure 4). Quantitative real-time PCR (qRT-PCR) indicated that the relative expression level of *TaGS5-A1b* was higher than that of *TaGS5-A1a* in all six stages.

## Discussion

Wheat yield is usually the most important trait for wheat breeders over the long-term improvement of bread wheat as it reflects the culmination of all the processes of vegetative and reproductive growth, as well as their interactions with the edaphic and aerial environments (Quarrie et al., 2006). Wheat yield greatly increased through continuous artificial selection; especially, improvement of lodge resistance sharply increased wheat production by taking advantage of dwarf genes (*Rht-B1* and *Rht-D1*) in the 1960s and 1970s (Peng et al., 1999), which resulted in the worldwide “Green Revolution.” However, wheat yield has not significantly improved in the past decade because of the increasingly narrow genetic basis of wheat germplasm in current wheat breeding program. Therefore, discovering new gene germplasms with relatively superior agronomic traits is markedly beneficial in improving wheat yield. In this study, we cloned *TaGS5* gene from bread wheat and identified two alleles at the *TaGS5-A1* locus. Furthermore, allelic variation of *TaGS5-A1* gene was intimately associated with agronomic traits. However, this association showed significant difference in landraces and modern cultivars. The larger differences of the agronomic traits between *TaGS5-A1a* and *TaGS5-A1b* genotypes in the landraces suggested that *TaGS5* gene played an important role in modulating yield-related traits in the landraces. The differences may possibly be caused by more relatively superior genes related to yield traits gathered in the modern Chinese cultivars after strong artificial selection in view of agronomic traits (Wang et al., 2012a).

Chinese landraces usually show higher plant height, easy lodging, and lower yield compared with modern cultivars. However, landraces are precious germplasm for their aspects as almost unselected populations, including yield-related traits. The preferred *TaGS5-A1b* genotype for high TKW was more prevalent in modern cultivars than in landraces, indicating that *TaGS5-A1b* genotype underwent very strong positive selection in modern Chinese wheat breeding. Interestingly, a paradox seemed to occur on the association between *TaGS5-A1* gene and kernel length, i.e., wheat landraces with *TaGS5-A1b* allele possessed longer kernel length than that of landraces with *TaGS5-A1a* allele, but the comparison result was the exact opposite in the modern Chinese cultivars. Kernel length increase in the landraces possibly resulted from improvement of lodging resistance because cultivars with *TaGS5-A1b* allele showed 10–20% lower plant height than cultivars with *TaGS5-A1a* allele.

Chinese wheat is mainly planted in 10 agro-ecological zones, which are further divided into 26 sub-zones. Winter, facultative, and spring wheats are sown both in autumn and spring. Of all agro-ecological zones, the Yellow and Huai wheat production regions, which cover all of Henan and parts of Shandong, Hebei, Shanxi, Anhui, and Jiangsu Provinces, are the most important and largest wheat production zone with 60–70% of the total harvested area and the total wheat production. Successful utilization of dwarfing genes (*Rht1, Rht2*, and *Rht8*) and the 1B/1R translocation made significant contributions to yield improvement in the wheat-producing region of the Yellow and Huai Valleys, as indicated by Zhou et al. (2007). However, yield increase has been slowing down in recent years because of homogenization of parents in wheat breeding program of this region. The germplasm in this study was from the Yellow and Huai wheat regions. Most of the germplasms were currently popular cultivars or advanced lines, and some used to play the important role as parents in wheat breeding in the Yellow and Huai wheat regions. Therefore, uncovering yield-related genes and understanding their association with agronomic traits could provide useful information for further improvement of yield in the wheat breeding program of the Yellow and Huai wheat regions of China.

In this study, *TaGS5* genes were physically mapped on the chromosome of 3A and 3D and were closely associated with kernel size, TKW, plant height, spike length, and internode length below spike. To date, previous studies indicated that almost all chromosomes of bread wheat harbored yield-related QTLs, including group 3 (Wang et al., 2009; Wu et al., 2012; Zhang et al., 2012; Cui et al., 2014; Liu et al., 2014). In particular, a number of QTLs in 3A and 3D chromosome were identified to control plant height, spike length, spike per plant, kernel number per spike, kernel weight per spike, kernel length, kernel width, and TKW (Cui et al., 2014; Liu et al., 2014). These QTLs account for 5.26–15.82% of the phenotypic variation. A TKW-related QTL was previously mapped to be linked with Barc356 on chromosome 3A in bread wheat (Cui et al., 2014). Liu et al. (2014) indicated that a QTL in the interval of Barc324-Xwmc428 on chromosome 3A was detected to be associated with plant height in five environments. In this study, *TaGS5-A1* gene was intimately linked to Barc356 and Barc324 in the F10 RIL population. However, more research is required to further evaluate the apparent association of the *TaGS5* loci with agronomic traits.

The great influence of single-nucleotide change on genes has been studied for many years; a single-nucleotide change could result in a strong influence on genes and further cause apparent change of phenotype (Slade et al., 2005; Uauy et al., 2009; Jiao et al., 2010; Kurowska et al., 2011; Rawat et al., 2012; Chen et al., 2014; Dabhi and Mistry, 2014; Sharp and Dong, 2014). Jiao et al. (2010) indicated that a point mutation in the third exon of *OsSPL14* gene generated an “ideal” rice plant with a reduced tiller number, increased lodging resistance, and enhanced kernel yield. In the present study, *TaGS5-A1* gene exhibited a single-nucleotide change belonging to non-synonymous mutation in the sixth exon, resulting in the change of kernel size, and other agronomic traits. Evidence also suggested that non-synonymous mutations could directly or indirectly destabilize the amino acid interactions and hydrogen bond networks and finally affecting protein function (Dabhi and Mistry, 2014). Therefore, the discovery of the *TaGS5-A1b* allele will be useful for the study of interaction relationship of the yield-related genes because the non-synonymous mutation in the FBG domain of *TaGS5-A1* gene might change the structure of the *TaGS5-A1* protein.

## Materials and methods

### Plant materials

A total of 363 wheat cultivars were planted in 2012–2013, 2013–2014, and 2014–2015 cropping seasons at the experimental field of Zhengzhou Scientific Research and Education Center of Henan Agricultural University (N34.9°, E113.6°). Accessions used in this study were composed of 41 landraces and 322 modern cultivars or advanced lines from the Yellow and Huai wheat regions of China. The field experimental design was performed with random complete block design with two replicates. Each plot contained four 200 cm long rows with 23 cm between neighboring rows and 10 cm between neighboring plants (Chen et al., 2013a,b). Test plots were managed according to local practices. The plant height, spike length, internode length below spike, and spikelet number of 10 spikes of each cultivar surveyed were investigated in the fields before the plants were harvested. After harvest, the average kernel number of 10 spikes of each cultivar surveyed, as well as the kernel length, kernel width, GL/GW ratio, and TKW of two plants of each cultivar surveyed, was investigated using naturally dried materials.

An entire set of Chinese Spring nullisomic–tetrasomic lines and ditelosomic lines were used to map the *TaGS5* genes on chromosomes of Chinese Spring. Professor Jorge Dubcovsky from the University of California, Davis, kindly provided F_10_RIL population (UC 1110 × PI 610750) composed of 153 lines, which were used for further physical mapping of *TaGS5-A1* and analysis of association between *TaGS5-A1* alleles and kernel size. The RIL population was planted in October 2013 and was harvested in the June 2014 cropping season at Anyang (N36.1°, E114.5°) and Zhengzhou (N34.9°, E113.6°); the population grew well at both locations. No lodge occurred in the entire field experiment. Agronomic traits (e.g., plant height, spike length, spikelet number per spike, kernel length, kernel width, and TKW) were investigated as mentioned above.

### DNA extraction and polymerase chain reaction amplification parameters

The total genomic DNA of all wheat cultivars surveyed in this study was extracted using CTAB method (Chen et al., 2010). PCR amplifications were performed in a Bio-Rad S1000 or ABI 9700 thermal cyclers. The PCR reaction system with total volumes of 25 μL was composed of 0.5 μL of each primer (10 pmol/μL), 100 ng genomic DNA, 2.5 μL 10 × Taq buffer (Mg^2+^ plus), 0.5 μL dNTP (2.5 mM), and 1.25 U Taq DNA polymerases (Tiangen Biotech, Beijing Co., Ltd.). The PCR reaction procedure consisted of three stages. The first stage involved 94°C for 5 min. The second stage included 94°C for 30 s, annealing (55–59°C) for 30 s, and 72°C for 1–2 min, performing 35 cycles. The last stage was 72°C for 7 min. PCR products were analyzed and separated on 1.0–1.5% (w/v) agarose gels, stained with ethidium bromide, and visualized using UV light. The expected PCR products were purified using the SanPrep Column DNA Gel Extraction Kit (Shanghai Biological Technology Co., Ltd.). All purified PCR products were ligated into pMD18-T vector (TaKaRa Biotechnology Co., Ltd., Dalian) and were transformed into cells of the *Escherichia coli DH-5*α strain. Plasmids containing targeted fragments were extracted using Plasmid Rapid Isolation Kit (Shanghai Biological Technology Co., Ltd.) and were sequenced for 12 clones for each sample using Shanghai Sangon Biotech Co., Ltd. The sequencing reliability of each subclone was checked using Chromas 2.4.3 (http://technelysium.com.au/?page_id=13) and FinchTV 1.5.0 (http://www.geospiza.com/Products/finchtv.shtml).

### Primer designing for cloning *TaGS5* genes in bread wheat

Based on the cDNA sequence of *OsGS5* gene (JN256056 and JN256055) in rice, several new contigs were obtained by alignment in a durum wheat transcriptome (kindly provided by Professor Jorge Dubcovsky) and a hexaploid wheat transcriptome of Yunong 201 (unpublished data in our lab). In addition, several wheat ESTs were gained by blasting cDNA sequence of *OsGS5* in NCBI database. The collected wheat sequences and the cDNA sequence of *OsGS5* were aligned and assembled using DNAMAN Version 6.0 software (http://www.softlandsl.com/free/dnaman+6+full.html). Based on the assembled sequence, four pairs of primer sets (TaGS5_−_P1, TaGS5_−_P2, TaGS5_−_P3, and TaGS5_−_P4; Table 1) were designed to amplify the full length of the cDNA sequence of GS5 gene in Chinese Spring. Finally, a primer set (*Ta*GS5_−_P1) successfully amplified full-length *TaGS5* cDNA sequence in Chinese Spring. Based on the obtained cDNA sequence of *TaGS5* gene, five new primer sets (*Ta*GS5_−_P5, *Ta*GS5_−_P6, *Ta*GS5_−_P7, *Ta*GS5_−_P8, and *Ta*GS5_−_P9; Table 1) were designed to amplify *TaGS5* genomic DNA sequence. All primers (Table 1) in this study were designed using Premier Primer 3.0 (http://primer3.ut.ee/) and Premier Primer 5.0 and were synthesized by Shanghai Biological Technology Co., Ltd. (http://www.sangon.com/).

### Development of CAPS marker for identification of *TaGS5-A1* alleles

Genomic-specific primer set TaGS5_−_P14 (Table 1) was designed for the development of CAPS marker to distinguish T/G at the six exons of the *TaGS5-A1* gDNA sequence. Amplification fragments were purified and were digested by *Bbv1* restriction enzyme (New England Biolabs Co., Ltd). The reaction system of *Bbv1* restriction enzyme with total volumes of 50 μL were composed of 5 μL 10 × NEB buffer II, 1 μL *Bbv1* restriction enzyme, 2 μL PCR production, and 42 μL dd H_2_O. The mixed solutions were stably kept at 37°C for 40 min in water bath tank (HH-S4A Double-row4-hole). Finally, the digested products were separated on 1.5% (w/v) agarose gels.

### Quantitative real-time PCR analysis of *TaGS5-A1a* and *TaGS5-A1b* genotypes

The total RNA of three-leaf seedlings was extracted using Trizol reagent and was reverse-transcripted to cDNA through PrimeScript RT reagent kit with gDNA Eraser (TaKaRa Biotechnology Co., Ltd, Dalian), following the kit instruction. A pair of specific primers (TaGS5_−_P1, Table 1) was used by reverse transcriptional PCR. The PCR products were linked to pGEM18-T vector by pGEM18-T simple vector kit (TaKaRa: D103A). *E. coli* (*Ta*GS5-cDNA-T) were cultured for 6–8 h at 37°C and sequenced by Shanghai Biological Technology Co., Ltd.

Gene-specific primers TaGS5-P15 and TaGS5-P16 were designed to examine the expression levels of *TaGS5-A1a* and *TaGS5-A1b* genotypes. *18s* gene was selected as internal control with the primer 18s (Table 1). The expression profiles of *TaGS5-A1a* and *TaGS5-A1b* genotypes were measured using the cDNA samples from seeds of different developmental stages (i.e., 7d, 14d, 21d, 28d, 35d, and 42d after anthesis) of cultivars Zhengmai 9023 with *TaGS5-A1a* and Yunong 211 with *TaGS5-A1b*. Bio-Rad iQ5 Sequence Detection System (Applied Biosynthesis, CA, USA) with SYBR Premix Ex TaqII (TaKaRa Biotechnology Co., Ltd, Dalian) was used to perform qRT-PCR. The PCR conditions consisted of an initial denaturation step for 2 min at 94°C, followed by 40 cycles of 10 s at 95°C, 10 s at 56°C, 20 s at 72°C, and a final extension of 10 min at 72°C. The 2^−ΔΔCT^ method was used to normalize and calibrate transcript values relative to the endogenous *18s* control. Six independent samples with triplicate repeats were analyzed.

### QTL mapping and statistical analyses

Physical mapping and QTL analysis of *TaGS5-A1* genes in the F_10_ population were performed using IciMapping 4.0 software (http://www.isbreeding.net/). Detailed genetic map of the F_10_ population was in Lowe et al. (2011) and we only used markers from 3A chromosome to map traits surveyed. QTL scanning was performed using inclusive composite interval mapping through stepwise regression by considering all marker information on chromosome 3 using 2.5 as the threshold LOD score (Meng et al., 2015).

Analysis of variance was conducted using PROC MIXED in the Statistical Analysis System (SAS Institute Inc., 2000) with genotype classes as categorical variables to derive the means of agronomic traits for each class and to test the significant level for the two classes. The differences of agronomic traits among different genotypes were tested by least significant range multiple comparisons.

## Author contributions

FC designed the project. SW performed experiment. XZ and DC investigated agronomic traits. FC and SW wrote the paper.

### Conflict of interest statement

The authors declare that the research was conducted in the absence of any commercial or financial relationships that could be construed as a potential conflict of interest.
