# Supplementary material for: A Single-Nucleotide Polymorphism of TaGS5 Gene Revealed its Association with Kernel Weight in Chinese Bread Wheat
Source: Front Plant Sci. 2015 Dec 23;6:1166. doi: 10.3389/fpls.2015.01166 (PMC4688388; doi:10.3389/fpls.2015.01166)
Supplement: Supplementary file 1 [file Image1.PDF]

TaGS5-A1a	ATGGCGGCAGCGGCGAACCAAGACAAGCAGCGGCGGCAGCTCATCCTTGCTCTCTTTCTCCTCGTCTTGGCCTCCCAGAG	80
TaGS5-A1b	ATGGCGGCAGCGGCGAACCAAGACAAGCAGCGGCGGCAGCTCATCCTTGCTCTCTTTCTCCTCGTCTTGGCCTCCCAGAG	80
TaGS5-D1	ATGGCGGCAGCGGCGAACCAAGACAAGCAGCGGCGGCAGCTCATCCTTGCTATCTTTCTCCTCGTCTTGGCCTCCCAGAG	80
TaGS5-A1a	TTGGCGCTGCTGCTCTGCAGGTTACGGTAGTGGGCAGGAAGCTGATAGGGTGGTCTTCCTCCCCGGTCAGCCAAGGAGCC	160
TaGS5-A1b	TTGGCGCTGCTGCTCTGCAGGTTACGGTAGTGGGCAGGAAGCTGATAGGGTGGTCTTCCTCCCCGGTCAGCCAAGGAGCC	160
TaGS5-D1	TTGGCGCTGCTGCTCTGCAGGTTACGGTAGTGGGCAGGAAGCTGATAGGGTGGTCTTCCTCCCCGGTCAGCCAAGGAGCC	160
TaGS5-A1a	CGCAGGTCTCTCAGTTCGCCGGGCATGTCACCGTGAACAAGCGGAATGGGAGGGCGCTCTTCTACTGGTTCTTCGAGGCT	240
TaGS5-A1b	CGCAGGTCTCTCAGTTCGCCGGGCATGTCACCGTGAACAAGCGGAATGGGAGGGCGCTCTTCTACTGGTTCTTCGAGGCT	240
TaGS5-D1	CGCAGGTCTCTCAGTTCGCCGGGCATGTCACCGTGAACAAGCGGAATGGGAGGGCGCTCTTCTACTGGTTCTTCCAGGCT	240
TaGS5-A1a	CAGTCACAGCCCTCGCACAAACCTCTCCTGCTCTGGCTCAATGGAGGTTTCTTACTTTGCACTTATTCACTTGTCTGTTC	320
TaGS5-A1b	CAGTCACAGCCCTCGCACAAACCTCTCCTGCTCTGGCTCAATGGAGGTTTCTTACTTTGCACTTATTCACTTGTCTGTTC	320
TaGS5-D1	CAGTCACAGCCCTCGCACAAGCCTCTCCTGCTCTGGCTCAATGGAGGTTTCTTACTTTGCACTTATTCACTTGTCTGTTC	320
TaGS5-A1a	TCTCTCTTTTATTTTACCTCACTTATTTCAGGAAATGTTTTGTAGAGATTATCTTGTGTTCCTCTCCTCGCGGTTCAGTA	400
TaGS5-A1b	TCTCTCTTTTATTTTACCTCACTTATTTCAGGAAATGTTTTGTAGAGATTATCTTGTGTTCCTCTCCTCGCGGTTCAGTA	400
TaGS5-D1	TCTCTCTTTTATTTTACCTCACTTATTTCAGGAAATGTTTTGTAGAGATTATCTTGTGT.....TCCTCGCGGTTCAGTA	395
TaGS5-A1a	ATAATTCGGTTGCTACTTGGGGTGAAAAATAAACAACTTTTTTGTTCCCTGCACATGCATGCAGGACCAGGCTGCTCATC	480
TaGS5-A1b	ATAATTCGGTTGCTACTTGGGGTGAAAAATAAACAACTTTTTTGTTCCCTGCACATGCATGCAGGACCAGGCTGCTCATC	480
TaGS5-D1	ATAATTCGGTTGCTACTTGGGGTGAAAAATAAACAACTTTTTTGTTCCCTGCACATGCATGCAGGACCAGGCTGCTCATC	475
TaGS5-A1a	AGTTGGATACGGAGCTGCTTCTGAGCTAGGACCTCTCAGGGTGAGAAGATTTGCAGCAGGGCTCGAGTTCAACAAGTTTG	560
TaGS5-A1b	AGTTGGATACGGAGCTGCTTCTGAGCTAGGACCTCTCAGGGTGAGAAGATTTGCAGCAGGGCTCGAGTTCAACAAGTTTG	560
TaGS5-D1	AGTTGGATACGGAGCTGCTTCTGAGCTAGGACCTCTCACGGTGAGCAGATTTGCAGCAGGGCTCGAGTTCAACAAGTTTG	555
TaGS5-A1a	CATGGAACAAAGGTGTTGCAGCTTGCAAGCATTGCACTTGTGCTAACTTATTATGAATTTATGATTATCCCAACGGTTCT	640
TaGS5-A1b	CATGGAACAAAGGTGTTGCAGCTTGCAAGCATTGCACTTGTGCTAACTTATTATGAATTTATGATTATCCCAACGGTTCT	640
TaGS5-D1	CATGGAACAAAGGTGTTGCAGCTTGCAAGCATTGCACTTGTGCTAACTTATTATGAATTTATGATTATCCCAACGGTACT	635
TaGS5-A1a	ACCTACGT..AAAAAAATCTGAATATTGGTATTGTTCTCGATTGACTCTTGCCAGAAGCCAACCTGCTCTTCGTGGAGTC	718
TaGS5-A1b	ACCTACGT..AAAAAAATCTGAATATTGGTATTGTTCTCGATTGACTCTTGCCAGAAGCCAACCTGCTCTTCGTGGAGTC	718
TaGS5-D1	ACCTACGTAAAAAAAAATCTGAATATTGGTATTGTTCTCGATTGACTCTTGCCAGAAGCCAACTTGCTCTTCGTGGAGTC	715

TaGS5-A1a	CCCTGTTGGGGTTGGCTTCTCCTACACCAACACATCCTCTGACCTCACCAACCTCAATGATGATTTTGTAGGTAACTGAG	798
TaGS5-A1b	CCCTGTTGGGGTTGGCTTCTCCTACACCAACACATCCTCTGACCTCACCAACCTCAATGATGATTTTGTAGGTAACTGAG	798
TaGS5-D1	TCCTGTTGGGGTTGGCTTCTCCTACACCAACACATCCTCTGACCTCACCAACCTCAATGATGATTTTGTAGGTAACTGAG	795
TaGS5-A1a	TATTCTTTTCGGGAAGTGTGCTTGCTTTTCATAACTGTTTGAGGCGTTTTGAAATGTTCATCAATTTCCATTTTCTCTTT	878
TaGS5-A1b	TATTCTTTTCGGGAAGTGTGCTTGCTTTTCATAACTGTTTGAGGCGTTTTGAAATGTTCATCAATTTCCATTTTCTCTTT	878
TaGS5-D1	TATTCTTTTCGGGAAGTGTGCTTGCTTTTCATAACTGTTTGAGGCGTTTTGAAATGTTCATCAATTTCTATTTTCTCTTT	875
TaGS5-A1a	TGTATAGCTGAGGATACATATAATTTCCTGATTAATTGGTTCAAGAGGTTTCCGCAGTACAAGGACCGGGAATTCTATAT	958
TaGS5-A1b	TGTATAGCTGAGGATACATATAATTTCCTGATTAATTGGTTCAAGAGGTTTCCGCAGTACAAGGACCGGGAATTCTATAT	958
TaGS5-D1	TGTATAGCTGAGGATACATATAATTTCCTGATTAATTGGTTCAAGAGGTTTCCGCAGTACAAGGACCAGGAGTTCTATAT	955
TaGS5-A1a	CTCAGGAGAGAGCTATGCAGGTAAATGTTTACATCGACAAGACACTTGTTCTTCAGGCCTTGATTTCCAGAGTTGATCTT	1038
TaGS5-A1b	CTCAGGAGAGAGCTATGCAGGTAAATGTTTACATCGACAAGACACTTGTTCTTCAGGCCTTGATTTCCAGAGTTGATCTT	1038
TaGS5-D1	CTCAGGAGAGAGCTATGCAGGTAAATGTTTACATCAACAAGACACTT.TTCTTCAGGCCTTGATTTCCAGAGTTGATCTT	1034
TaGS5-A1a	AGTAACCTGCAAACTTAATTTTTTCAATATAGTTGTCCTACATGTCTGACAAAGTCGTAAAAA.TAATAAGTCAATCTGG	1117
TaGS5-A1b	AGTAACCTGCAAACTTAATTTTTTCAATATAGTTGTCCTACATGTCTGACAAAGTCGTAAAAA.TAATAAGTCAATCTGG	1117
TaGS5-D1	AGTAACCTGCAAACTTAAA.TTTTCAATATAGTTGTCCTACATATCTGACAAAGTCATAAAAAtTAATAAGTCGACCTGG	1113
TaGS5-A1a	CCTTTCAAATTAGGTTCTTTACCAATTCCTATTTATTTTAGTGTAAACCATAAATCATTAAATTTAAAGGAATTGGGCAA	1197
TaGS5-A1b	CCTTTCAAATTAGGTTCTTTACCAATTCCTATTTATTTTAGTGTAAACCATAAATCATTAAATTTAAAGGAATTGGGCAA	1197
TaGS5-D1	CCTTTCAAATTAGGTTCTTTACCAATTCCTATTTATTTTTGTGTAATCCATAAATCATTAAATTTAAATTAATTGGGCAA	1193
TaGS5-A1a	TAATTGATGTTAAAGCA...	1214
TaGS5-A1b	TAATTGATGTTAAAGCA...	1214
TaGS5-D1	TAACTGATGTTAAAGCATACTCCCTCCGTCCCAACTATAATAGCGGTTTTGACACTTTTTGAGCGTTTTTGACACTACAC	1273
TaGS5-A1a	....TAGATGGGAAATTTTGGTGAGAAAATTTTAAAATATTAATC	1255
TaGS5-A1b	....TAGATGGGAAATTTTGGTGAGAAAATTTTAAAATATTAATC	1255
TaGS5-D1	TAGTGTTAAAAACGCTCTTATATTTTGGGACGGAGGGAGTAGATGGGAAATGTTGGTGAGAACATTTTAAAATATTAATC	1353
TaGS5-A1a	CTGGCAAGTCATGATGCTCCTGTAGTTCAGAGGAAACTTAAAGGTGGGTAATATGCTCATCTCATTTTCCCCACCCATTC	1335
TaGS5-A1b	CTGGCAAGTCATGATGCTCCTGTAGTTCAGAGGAAACTTAAAGGTGGGTAATATGCTCATCTCATTTTCCCCACCCATTC	1335

TaGS5-D1 CTGGCAAGTCATGATGCTTCTGTAGTTCAGAGGAAACTTAATGGTGGATACTATGATCATCTCATTTTCCCCACCAATTC 1433

TaGS5-A1a CTGCCTACCTTCTTTTTGGCCAATTTTAGTGTTCGTTTGATCCCCTTTTGTCGAATTTTGTGACTCATTTTCTACAACAT 1415
TaGS5-A1b CTGCCTACCTTCTTTTTGGCCAATTTTAGTGTTCGTTTGATCCCCTTTTGTCGAATTTTGTGACTCATTTTCTACAACAT 1415
TaGS5-D1 CTGCCTACCTTCTTTTTGGCC.ATTTTAGTGTTCGTTTGACCCGCTTTTGTCTAATTTTGTGACTCATTTTCTACAACAT 1512

TaGS5-A1a ATTATCAAACAAGCACACAACACGGTTCAGCAACCTCAGATTGGGCCTACATTGCTATGTGTCTTGTAACCTGGGAGAAC 1495
TaGS5-A1b ATTATCAAACAAGCACACAACACGGTTCAGCAACCTCAGATTGGGCCTACATTGCTATGTGTCTTGTAACCTGGGAGAAC 1495
TaGS5-D1 ATTATCAAATAAGCACACAGCACAA....CACGGTTCAGCAACCTCAGATTGGGCCCTATGTGTCTTGTAACCTGGGAAAAA 1590

TaGS5-A1a AGAGGTTTACCATACCATGGAAACAAAGCAAAGTGAATAACATTTTGGTGGCATGGCCCTTTAGATTGGTTCCGGTATTC 1575
TaGS5-A1b AGAGGTTTACCATACCATGGAAACAAAGCAAAGTGAATAACATTTTGGTGGCATGGCCCTTTAGATTGGTTCCGGTATTC 1575
TaGS5-D1 CAGAGGTTTTT.....TGGAAGTACCATACCATGGAAACAAAGCAAAGTGAATAATATTTTGGTGGCATGGCCCTTTAG 1665

TaGS5-A1a CTGACATGTCCAATGGTCATATTAGACTTCTGTGATATTTCAGACATGGTGGAGCAAGAGATGCGCTGCAATTCTATTTT 1655
TaGS5-A1b CTGACATGTCCAATGGTCATATTAGACTTCTGTGATATTTCAGACATGGTGGAGCAAGAGATGCGCTGCAATTCTATTTT 1655
TaGS5-D1 ATTGGTTCTGGTATTCCTGACATATCAAATGGTCATATCAGACCTTTGT............................GATATTTCTGCAATTCCTTTT 1735

TaGS5-A1a TGTAGATCACTAGTGAAGAATTTTAAATTTTATACAGGTCATTATGTGCCACAACTAGCTGACCTTGTCTATGAGAGGAA 1735
TaGS5-A1b TGTAGATCACTAGTGAAGAATTTTAAATTTTATACAGGTCATTATGTGCCACAACTAGCTGACCTTGTCTATGAGAGGAA 1735
TaGS5-D1 TGTATACCACTGACGAAGAATTTTAAATTTTATACAGGTCATTATGTGCCACAACTAGCTGACCTTGTCTATGAGAGGAA 1815

TaGS5-A1a CAAAGACAAGAAGGCCAGCACATACATCAACTTTAAAGGGTTCATTGTGAGTATATAATTTGATAGCTTCTGTTGTTTAT 1815
TaGS5-A1b CAAAGACAAGAAGGCCAGCACATACATCAACTTTAAAGGGTTCATTGTGAGTATATAATTTGATAGCTTCTGTTGTTTAT 1815
TaGS5-D1 CAAAGAtAAGAAGGCCAaCACATACATCAACTTTAAAGGGTTCATTGTGAGTATATAATTTGATAGCTTCaGTTGTTTAT 1895

TaGS5-A1a ATTGCTGGTTCGGCCAGTGGCCTTGTTCATTCTTGTTTGAAAATGGGATTACATAATGTATTGTTTTTTATAGTTTATAA 1895
TaGS5-A1b ATTGCTGGTTCGGCCAGTGGCCTTGTTCATTCTTGTTTGAAAATGGGATTACATAATGTATTGTTTTTTATAGTTTATAA 1895
TaGS5-D1 ATTGCTGGTTTGGCCAGTGGCCTTGTTCATTCTTGTTTGAAAATGGGATGACATAGTTTATTCTTTTTTAGAGTTGATAA 1975

TaGS5-A1a CTTCGAAATAATTCATGTATTTCCTGGGATGATTTAGATGAAAAATATTGCATACTTATTTCTGTGAAAATTACAACAAT 1975
TaGS5-A1b CTTCGAAATAATTCATGTATTTCCTGGGATGATTTAGATGAAAAATATTGCATACTTATTTCTGTGAAAATTACAACAAT 1975
TaGS5-D1 CTTTGAAATAATTCATGTATTTCCTGGGATGATTTACATGAAAAACATTGCATACTTATTTCTGTGAAAATTAAAACAAT 2055

TaGS5-A1a CTTGGGATTTGTTAACCTTCCGATGAACTTCCATTTTTTTCTATAAATGTATATATTGCCTAGGTTTTCTGTGGATAGTT 2055

TaGS5-A1b CTTGGGATTTGTTAACCTTCCGATGAACTTCCATTTTTTTCTATAAATGTATATATTGCCTAGGTTTTCTGTGGATAGTT 2055
TaGS5-D1 CCTGGGATTTGTTAACCTTCTGATGAACTTCCCTTTTTTTTTATAAATGTATATATTGCCTAGGTTTCCTGTGGATAGTT 2135

TaGS5-A1a TGAAATTTTATAACATCATAACATTTATATTGGCCCAAATGTAGGTTGGAAATCCATTAACTGATGATTATTATGACTCG 2135
TaGS5-A1b TGAAATTTTATAACATCATAACATTTATATTGGCCCAAATGTAGGTTGGAAATCCATTAACTGATGATTATTATGACTCG 2135
TaGS5-D1 TGAAATTTTATAACATCATAACATTTATATTGGCCC.AATGTAGGTTGGCAATCCATTAACTGATGATTACTATGACTCG 2214

TaGS5-A1a AAGGGACTGGCCGAATATGCTTGGAGCCATGCAGTTGTATCAGATGAAGTTTATGATCAGATAAAAAAGAATTGTGATTT 2215
TaGS5-A1b AAGGGACTGGCCGAATATGCTTGGAGCCATGCAGTTGTATCAGATGAAGTTTATGATCAGATAAAAAAGAATTGTGATTT 2215
TaGS5-D1 AAGGGACTGGCCGAATATGCTTGGAGCCATGCAGTTGTATCAGATGAAGTTTACGATCGCATAAAAAAGGATTGTGATTT 2294

TaGS5-A1a TAGAGCCTCAAACTGGACCGATGATTGCAACAAAGCCATGAACACCATCTATGGACAGTACCAGTTGATCGACATATACA 2295
TaGS5-A1b TAGAGCCTCAAACTGGACCGATGATTGCAACAAAGCCATGAACACCATCTATGGACAGTACCAGTTGATCGACATATACA 2295
TaGS5-D1 TAGAGCCTCAAACTGGACCGATGATTGCAACAAAGCCATGAACACCATCTATGGACAGTACCAGTTGATCGACATATACA 2374

TaGS5-A1a ACATTTATGGGCCCAAGTGCAATCTTGGACAAACATCAGCAGCATCTGTTGTTGACAAAGCACTCAAATATAGTGACCAT 2375
TaGS5-A1b ACATTTATGGGCCCAAGTGCAATCTTGGACAAACATCATCAGCATCTGTTGTTGACAAAGCACTCAAATATAGTGACCAT 2375
TaGS5-D1 ACATTTATGCGCCCAAGTGCAATCTTGGACAAACATCAGCAGCATCCGTTGTTGAtAAAGCACTCGAATATAGTGACCAT 2454

TaGS5-A1a GTAAGGGACAGACAGTTTAAATTAGAACTTCTGCTTCTGAATTTCTAAGTATGTTAATTTCTTTGGCATGGTAGGAACCT 2455
TaGS5-A1b GTAAGGGACAGACAGTTTAAATTAGAACTTCTGCTTCTGAATTTCTAAGTATGTTAATTTCTTTGGCATGGTAGGAACCT 2455
TaGS5-D1 GTAAGGGACAGACAGTTTAAATTAGAACTTCTGCTTCTGAATTTCTAAGTATGTTAATTTCTTTGGCATGGTAGGAACCT 2534

TaGS5-A1a TTCAGGAGGAGGATTAGGTTGTTCTCAGGATACGATGAGTGCTACTCATCTTATGCTCAGGAGTACTTCAATAAGGCAGA 2535
TaGS5-A1b TTCAGGAGGAGGATTAGGTTGTTCTCAGGATACGATGAGTGCTACTCATCTTATGCTCAGGAGTACTTCAATAAGGCAGA 2535
TaGS5-D1 TTCAGGAGGAGAATTAGGTTGTTCTCGGGATATGATGAGTGCTACTCATCTTATGCTCAAGAGTACTTCAATAAGGCAGA 2614

TaGS5-A1a TGTGCAAAGGGCACTTCATGCAAATGTCAATGGGATGTTGCCTGGGAAATGGCAAGTTTGCAGGTGATTTCTGCAGATCT 2615
TaGS5-A1b TGTGCAAAGGGCACTTCATGCAAATGTCAATGGGATGTTGCCTGGGAAATGGCAAGTTTGCAGGTGATTTCTGCAGATCT 2615
TaGS5-D1 TGTGCAAAGGGCACTTCATGCAAATGTCAATGGGATGTTGCCTGGGAAATGGCAAGTTTGCAGGTGATTTCTGCAGAcCT 2694

TaGS5-A1a TTCCATTTTTTACCTATGAAATTTTAATCTAGTAATTACAACACTGAGTTGAATTATATCATGGGACATAGTTTAAAATA 2695
TaGS5-*A1b* TTCCATTTTTTACCTATGAAATTTTAATCTAGTAATTACAACACTGAGTTGAATTATATCATGGGACATAGTTTAAAATA 2695
TaGS5-D1 TTtCtTTTTTTATCTATGAAGTTTTAATCTAGTAGTTACAACTCTGAGTTGAATTATATCATGGGACATAGTTTAAAATA 2774

TaGS5-A1a	AGCGTTGCAATGTTAATATAGTTTTGATGTGATTCTACTTTGTGTCATTAACATTCTATCCAGGGACTCCAAACGAATGT	2775
TaGS5-A1b	AGCGTTGCAATGTTAATATAGTTTTGATGTGATTCTACTTTGTGTCATTAACATTCTATCCAGGGACTCCAAACGAATGT	2775
TaGS5-D1	AGCGTTGCAATGGTTATATAGTTTTGATGTGATTCTACTTTGTGTCATTAACATTCTATCCAGGGACTCCAAACAAATGT	2854
TaGS5-A1a	CAAATGGACAACTGCCGACTCAAATTTGGAAAATGCTCCAAAGAGATAATTTTACACGTGGAGAAGAAAT..........	2845
TaGS5-A1b	CAAATGGACAACTGCCGACTCAAATTTGGAAAATGCTCCAAAGAGATAATTTTACACGTGGAGAAGAAAT..........	2845
TaGS5-D1	CAAATGGAC.ACTACCGACTCAAATTCGGGAAATGCTCAAAAGAGATCATTTTACACGTGGAGAAGAAATTGAGCATGTT	2933
TaGS5-A1a	....TGAATTTGACTTATTTCCATTTTCATTTACCATGCAGTGACTCCATTCTGAAGTCGTACAACTTTTCGGTACTTTC	2921
TaGS5-A1b	....TGAATTTGACTTATTTCCATTTTCATTTACCATGCAGTGACTCCATTCTGAAGTCGTACAACTTTTCGGTACTTTC	2921
TaGS5-D1	AATATGAATTTGACTTATTTACATTTTCATTTACCATGCAGTGACTCCATTCTGAAGTCGTACAACTTTTCGGTACTTTC	3013
TaGS5-A1a	CATTCTACCAATATACTCCAAGCTCATCAAAGCAGGACTGAGAGTCTGGCTCTACAGGTGTATTTCTGCACCTTGTACCC	3001
TaGS5-A1b	CATTCTACCAATATACTCCAAGCTCATCAAAGCAGGACTGAGAGTCTGGCTCTACAGGTGTATTTCTGCACCTTGTACCC	3001
TaGS5-D1	CATTCTACCAATATACTCCAAGCTCATCAAAGCAGGACTGAGAGTCTGGCTCTACAGGTGTATTTCTGCACCTTGTACCC	3093
TaGS5-A1a	TACCACCATGTGTCAGTTCTCATATGCATTAAAAGTAGACACTGTTTGTTACTTCCATGTACTGATATTATCCTCCCAAA	3081
TaGS5-A1b	TACCACCATGTGTCAGTTCTCATATGCATTAAAAGTAGACACTGTTTGTTACTTCCATGTACTGATATTATCCTCCCAAA	3081
TaGS5-D1	TACCACCATGTGTCAGTTCTCATATGCATTCAAAGTAGACATTATTTGTTACTTCCATATACTGATATTATCCTCCCAAA	3173
TaGS5-A1a	TTACTTTCTGTAAGACGGTAACCTTAAAACACAATTTTGCTGTTCTCTGATGGCTTAAAAATCTTAGAATAGATGTTCTT	3161
TaGS5-A1b	TTACTTTCTGTAAGACGGTAACCTTAAAACACAATTTTGCTGTTCTCTGATGGCTTAAAAATCTTAGAATAGATGTTCTT	3161
TaGS5-D1	TTACTTTCGGTAAGACGGTGACCTTAAAACACAATTTTGCTGTTCTCTGATGGCTTAAAAATCTTAcAATAGATGTTCTT	3253
TaGS5-A1a	TTCATTGGCATGTTAGAATAGGTGCTATGTTGGTGTTCCGCAATCGGCAGTGACC..TATAATTTTGTGCATGCAGTGGA	3239
TaGS5-A1b	TTCATTGGCATGTTAGAATAGGTGCTATGTTGGTGTTCCGCAATCGGCAGTGACC..TATAATTTTGTGCATGCAGTGGA	3239
TaGS5-D1	TTCATTGGCATGTTAGAATAGGTGCTATGTTGGTGTTtCGGAATCGGCAGTGACCTTATAATTTTGTGGATGCAGTGGA	3333
TaGS5-A1a	GATGCGGATGGCAGGGTCCCAGTGATCGGGTCACGATATTGTGTGGAAGCACTTGGCTTGCCTATCAAGTCACAGTGGCA	3319
TaGS5-A1b	GATGCGGATGGCAGGGTCCCAGTGATCGGGTCACGATATTGTGTGGAAGCACTTGGCTTGCCTATCAAGTCACAGTGGCA	3319
TaGS5-D1	GATGCGGATGGCAGGGTCCCAGTGATCGGGTCACGATATTGTGTGGAAGCACTTGGCTTGCCTATCAAGTCACAGTGGCA	3413
TaGS5-A1a	ACCATGGTACCTGAACAAACAGGTCAGAAATTCTGAACAATCAAGTGAACATTCTCTGGGGCTACAATGATACTGCTAGT	3399
TaGS5-A1b	ACCATGGTACCTGAACAAACAGGTCAGAAATTCTGAACAATCAAGTGAACATTCTCTGGGGCTACAATGATACTGCTAGT	3399
TaGS5-D1	ACCATGGTACCTGAACAAACAGGTCAGAAATTCTGAACAATCAAGTGAACATTCTCTGGGGCTACAATGATACTGCTAGT	3493

TaGS5-A1a	TAAGTTAGTTTCTGAATGCAATCTAGACTCCACTGGCCCTGACGAGAGAGACATTTGCACTGTAGGTTGCTGGAAGATTT	3479
TaGS5-A1b	TAAGTTAGTTTCTGAATGCAATCTAGACTCCACTGGCCCTGACGAGAGAGACATTTGCACTGTAGGTTGCTGGAAGATTT	3479
TaGS5-D1	TAAGTTAGTTTATGAACGGAATCTAGACTCCACTGGCCCTGACGAGAGAGACATTTGCACTGTAGGTTGCTGGAAGATTT	3573
TaGS5-A1a	GTGGAGTACCATGGTATGACCATGGTGACAATAAGAGGGGCTGGCCACTTGGTACCCCTCAACAAGCCCACAGAAGGGAT	3559
TaGS5-A1b	GTGGAGTACCATGGTATGACCATGGTGACAATAAGAGGGGCTGGCCACTTGGTACCCCTCAACAAGCCCACAGAAGGGAT	3559
TaGS5-D1	GTGGAGTACCATGGTATGACCATGGTGACAATAAGAGGGGCTGGCCACTTGGTACCCCTCAACAAGCCCACAGAAGGGAT	3653
TaGS5-A1a	AGCGCTGATCGACACATTCCTTCTTGGTAAACAGCTTCCTACACACCGATGA	3611
TaGS5-A1b	AGCGCTGATCGACACATTCCTTCTTGGTAAACAGCTTCCTACACACCGATGA	3611
TaGS5-D1	AGCGCTGATCGACACATTCCTTCTTGGTAAACAGCTTCCTACACACCGATGA	3705

Fig. S1 Full alignment of *TaGS5-A1a*, *TaGS5-A1b and TaGS5-D1* genomic DNA sequence in bread wheat
